# Supplementary material for: A Rho-actin signaling pathway shapes cell wall boundaries in Arabidopsis xylem vessels
Source: Nat Commun. 2019 Jan 28;10:468. doi: 10.1038/s41467-019-08396-7 (PMC6349933; doi:10.1038/s41467-019-08396-7)

Supplementary Information

A Rho-actin signaling pathway shapes cell wall boundaries in Arabidopsis xylem vessels

Yuki Sugiyama, Yoshinobu Nagashima, Mayumi Wakazaki, Mayuko Sato,
Kiminori Toyooka, Hiroo Fukuda, Yoshihisa Oda

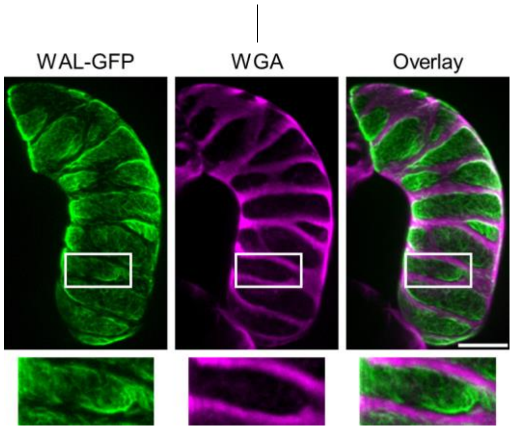

Supplementary Figure 1. WAL localized to the boundary of secondary cell wall pits. Localization of WAL-GFP (*LexA:WAL-GFP*) in a cultured xylem cell. Secondary cell walls are labeled with fluorescent wheat germ agglutinin (WGA). The lower panels show magnification of the boxed region in the upper panels. Bar = 10 µm.

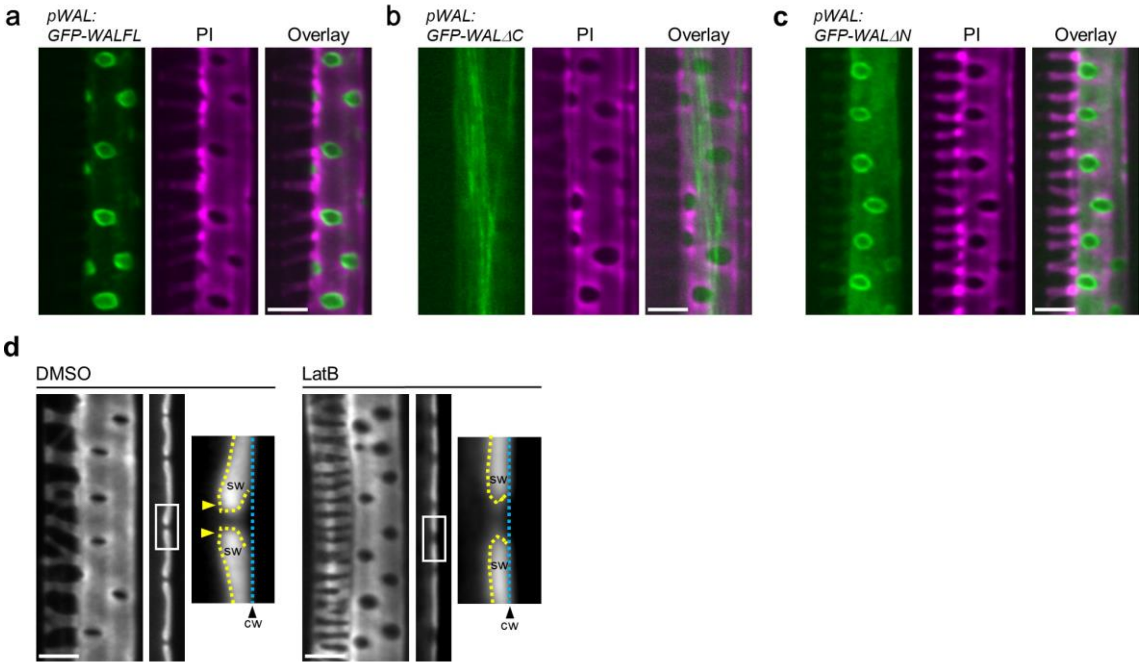

Supplementary Figure 2. WAL is recruited to the pits via its C-terminus.

(a–c) Localization of GFP-fused WAL (a), WALΔC (b), and WALΔN (c) in metaxylem vessel cells in roots of *wal* plants. Cell walls were stained with PI.

(d) Xylem vessels in roots treated with (LatB) or without (DMSO) 3 μM latrunculin B for 2 days. Secondary cell walls were stained with PI. The cortex (left) and mid-plane (center) from different cells are shown. The boxed area is magnified in the right panel. SW and CW indicate secondary cell walls and primary cell walls, respectively. Yellow arrowheads indicate bordered cell walls. Bars = 5 μm.

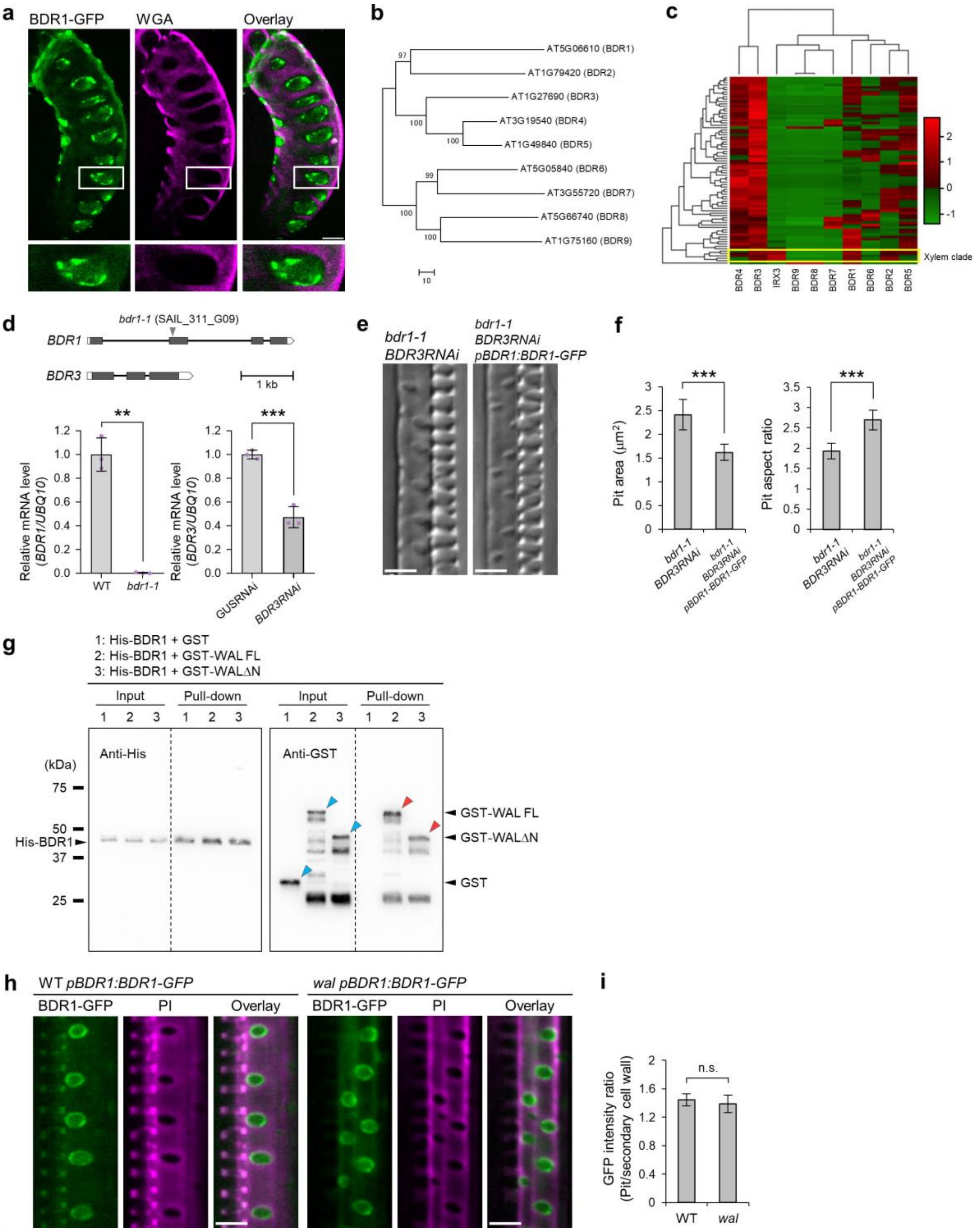

Supplementary Figure 3. BDR1 interacts with WAL at pit boundaries.

(a) Localization of BDR1-GFP (*LexA:BDR1-GFP*) in cultured xylem cells. Secondary cell walls are labeled with fluorescent WGA. The lower panels show magnification of the boxed regions in the upper panels.

(b) Phylogenetic tree of Arabidopsis BDR family. Branch lengths represent the number

of substitutions per site.
(c) Clustering analysis of *BDR* genes based on their expression patterns. The yellow box indicates the xylem vessel clade where the *IRX3* gene, encoding the xylem-specific cellulose synthase subunit [34], is active. The xylem vessel clade includes the axes of the first elongated internode and silique pods, where secondary cell walls are actively formed.
(d) Amount of *BDR1* and *BDR3* mRNA in *bdr1* (SAIL_311_G09) and *BDR3RNAi* plants, respectively. Values are mean ± s.d. (n = 3), **p < 0.01 (Student *t* test), ***p < 0.001 (Student *t* test).
(e) DIC of *bdr1-1 BDR3 RNAi* and *bdr1-1 BDR3 RNAi pBDR1:BDR1-GFP* plants.
(f) Surface area and aspect ratios of secondary cell wall pits. Values are mean ± s.d. (n > 250 pits), ***p < 0.001 (ANOVA with Scheffe test).
(g) Pull-down assay between BDR1 and WAL. His-BDR1 incubated with GST, GST-WAL FL, or GST-WALΔN was immobilized with Ni Sepharose resin. After washing with buffer, eluate was subjected to western analysis.
(h) Localization of BDR1-GFP (*pBDR1:BDR1-GFP*) in metaxylem vessel cells in roots of wild-type and *wal* plants.
(i) Intensity ratio of BDR1-GFP (inside of pit/outside of pit). Values are mean ± s.d. (n > 120), n.s., not significant (Student *t* test).
Bars = 10 µm (a) and 5 µm (e and h).

Source data are provided as a Source Data file.

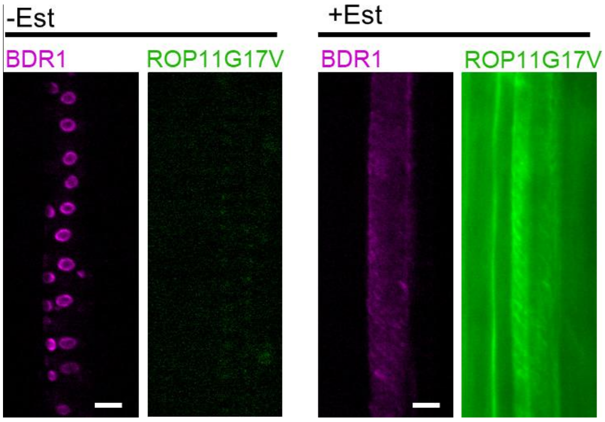

Supplementary Figure 4. ROP11 regulates localization of BDR1.

BDR1-tagRFP (*pBDR1:BDR1-tagRFP*) and GFP-ROP11G17V (*LexA:GFP-ROP11G17V*) in roots 2 days after treatment with (+Est) or without (-Est) estrogen. Bars = 5 µm.

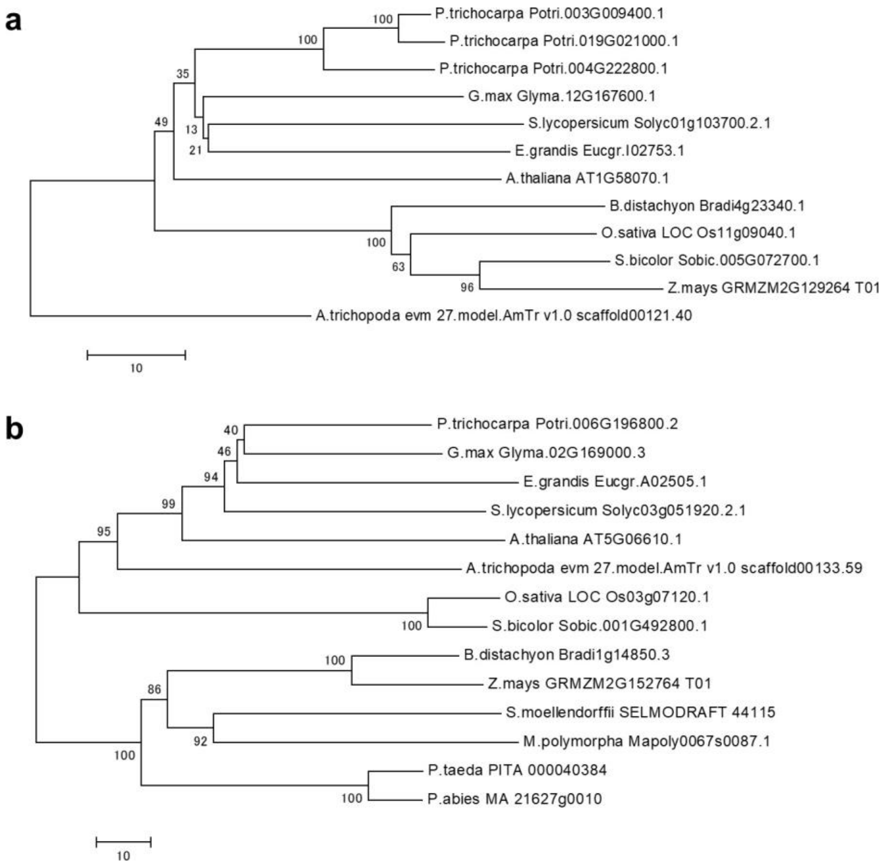

Supplementary Figure 5. Phylogenetic trees of WAL and BDR.

(a and b) Phylogenetic trees of WAL (a) and BDR (b) constructed using the neighbor-joining method. Branch lengths represent the number of substitutions per site. The closest member to AtBDR1 is listed from each species.

Supplementary Table 1. Primers

No.	Gene name	AGI code	Forward (5'-sequence-3')	Reverse (5'-sequence-3')	Note
1	WAL	At1g58070	CACCATGGATATAGAAGAGATGGAAGGAAATAATC	AAAGTGCAGGGTGGAAGAGG	
2	pWAL:WAL	At1g58070	CACCCTCTGCTTAAGACGTTGTGG	AAAGTGCAGGGTGGAAGAGG	
3	pWAL:GFP-WAL_1	At1g58070	TAAAAGGGTGGGCGCGCCGAC	GGAGGAACTATAGATAGAGAAGAATG	Inverse PCR
4	pWAL:GFP-WAL_2	GFP, At1g58070	TATAGTTCCTCCATGGTGAGCAAGGGCGAG	CCCACCCTTTTAAAAGTGCAGGGTGGAAG	In-Fusion with no. 3 fragment
5	WAL (1–300 bp)	At1g58070	CACCATGGATATAGAAGAGATGGAAGGAAATAATC	GCTAGGACTAGCAATAGGACTTACCAATTTAGC	
6	WAL (1–600 bp)	At1g58070	TAAAAGGGTGGGCGCGCCGAC	TTTTGTGTCCTTGAAGCTAACTG	Inverse PCR
7	WAL (301–855 bp)	At1g58070	ATAAGCCAGACCGCTTCTTCATCTTCTTC	CATGGTGAAGGGGGCGGC	Inverse PCR
8	pMIDD1	At3g53350	CACCTGCATAATCATCATCGCATGG	TTTTGGAGTTTAAAAACCTTTTCTTTTGGGG	In-Fusion with Lifeact vector
9	wal (T-DNA check)	At1g58070	CGGTGTTGCGAGTTACCTG	GTCCTAGCATCAGCCAGACTG	
10	WAL (Real time PCR)	At1g58070	GAGGAGGAAAGAGCTGATGAGG	AGTGTTCGCAAAATACCCATC	
11	UBQ10 (Real time PCR)	At4g05320	GAAGTTCAATGTTTCGTTTCATGT	GGATTATACAAGGCCCCAAAA	
12	BDR1	At5g06610	CACCATGCAGAGATTAGCTCCATTGATG	TGTATTGTAGTTTGGTAAATGTTTTTGAGC	
13	pBDR1(1700):BDR1	At5g06610	CACCGTGAATCTTGAGGTTTCTTTTATTAC	TGTATTGTAGTTTGGTAAATGTTTTTGAGC	BDR1-tagRFP
14	pBDR1(3000):BDR1	At5g06610	CACCTTAATAAATTACAGGTACCTTTTCTAAC	TGTATTGTAGTTTGGTAAATGTTTTTGAGC	BDR1-GFP
15	BDR1 (1–780 bp)	At5g06610	CACCATGCAGAGATTAGCTCCATTGATG	GTCTTCAAGGCAAATTAAGAGGCC	
16	BDR1 (301–1107 bp)	At5g06610	CACCATGATCAAAAACACTTTCGTGACCG	TGTATTGTAGTTTGGTAAATGTTTTTGAGC	
17	bdr1 (T-DNA check)	At5g06610	AGCACGTGACTAAGGCAAAAG	AAGCGTAGTGAATGCAAGAGTC	
18	bdr3-RNAi (1–400 bp)	At1g27690	CACCGGGAAGCTTGTGTGGAGAC	CCAGAGTGAGCTATCATGATACC	
19	BDR1 (Real time PCR)	At5g06610	CCTTAACCAGGATTCAGATACC	TCCATTTCTCTTCCATTCGAG	
20	BDR3 (Real time PCR)	At1g27690	GGCTACGCTTTTAAGGTTTGG	GTGCAATGTCCTAATTTTGGG	

Supplementary Reference

34 Taylor, N. G., Scheible, W. R., Cutler, S., Somerville, C. R. & Turner, S. R. The irregular xylem3 locus of Arabidopsis encodes a cellulose synthase required for secondary cell wall synthesis. Plant Cell **11**, 769-780 (1999).

Uncropped gel/blot images

Figure 2c

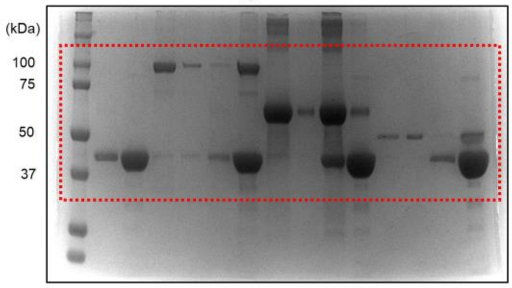

Supplementary Figure 3g

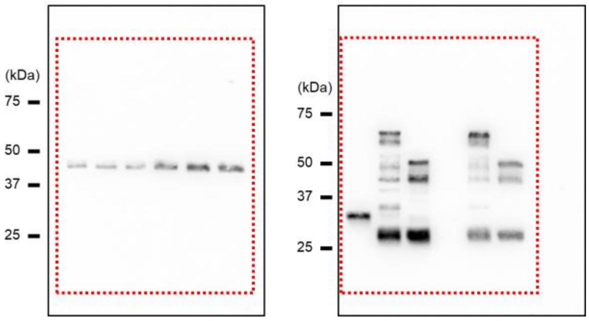

Supplement: Supplementary file 1 — Supplementary Information [file 41467_2019_8396_MOESM1_ESM.pdf]
